# Safety and Efficacy of Istaroxime in Patients With Acute Heart Failure: A Meta-Analysis of Randomized Controlled Trials

**DOI:** 10.7759/cureus.41084

**Published:** 2023-06-28

**Authors:** Saad Khalid Khan, Anurag Rawat, Zarghuna Khan, Ibrahim Reyaz, Vikash Kumar, Saima Batool, Rambabu Yadav, Shamsha Hirani

**Affiliations:** 1 Medicine, Army Medical College, Rawalpindi, PAK; 2 Interventional Cardiology, Himalayan Institute of Medical Sciences, Dehradun, IND; 3 Internal Medicine, Rehman Medical Institute, Peshawar, PAK; 4 Internal Medicine, Christian Medical College and Hospital Ludhiana, Punjab, Ludhiana, IND; 5 Medicine, Shaheed Zulfiqar Ali Bhutto Medical University, Islamabad, PAK; 6 Internal Medicine, Hameed Latif Hospital, Lahore, PAK; 7 Medicine, Ganeshmansingh Hospital, Lalitpur, NPL; 8 Cardiology, Baqai Hospital, Karachi, PAK

**Keywords:** meta-analysis, efficacy, safety, acute heart failure, istaroxime

## Abstract

The aim of this study was to assess the efficacy and safety of istaroxime in patients with heart failure. Following the Preferred Reporting Items for Systematic Review and Meta-Analyses (PRISMA) guidelines, a search was conducted on the EMBASE and Medline databases to identify articles related to the safety and efficacy of istaroxime in patients with heart failure. The search covered the period from inception to May 31st, 2023, without any restrictions on the year of publication. The search strategy utilized relevant terms such as "istaroxime," "heart failure", "efficacy," and other related terms, along with their corresponding Medical Subject Headings (MeSH) terms. The outcomes assessed in this meta-analysis included the change in left ventricular ejection fraction (LVEF), E to A ratio (a marker of left ventricle function), cardiac index in L/min/m2, systolic blood pressure (SBP) in mmHg, left ventricular end-systolic volume (LVESV) in ml, and left ventricular end-diastolic volume (LVDSV) in ml. For safety analysis, gastrointestinal events and cardiovascular events were assessed. A total of three randomized controlled trials (RCTs) were included in this meta-analysis encompassing 211 patients with heart failure. Pooled analysis showed that istaroxime was effective in increasing LVEF (MD: 1.26, 95% CI: 0.91 to 1.62, p-value: 0.001), reducing E to A ratio (MD: -0.39, 95% CI: -0.60 to -0.19, p-value: 0.001), increasing cardiac index (MD: 0.22, 95% CI: 0.18 to 0.25, p-value: 0.001), reducing LVESV (MD: -11.84, 95% CI: -13.91 to -9.78, p-value: 0.001), reducing LVEDV (MD: -12.25, 95% CI: -14.63 to -9.87, p-value: 0.001) and increasing SBP (MD: 8.41, 95% CI: 5.23 to 11.60, p-value: 0.001) compared to the placebo group. However, risk of gastrointestinal events was significantly higher in patients receiving istaroxime compared to the placebo group (RR: 2.64, 95% CI: 1.53 to 4.57, p-value: 0.0005). These findings support the enhancement of heart function with istaroxime administration, aligning with previous clinical and experimental evidence.

## Introduction and background

Heart failure is a medical condition defined by a range of symptoms, such as shortness of breath, swelling in the ankles, and tiredness. It also includes observable signs like swelling in the extremities, increased pressure in the jugular veins, and crackling sounds in the lungs. The primary cause of heart failure is typically related to abnormalities in the structure and/or function of the heart, resulting in inadequate pumping capacity to meet the body's metabolic needs or excessive pressure within the heart [1].

As the global population ages, the occurrence and prevalence of heart failure (HF) are rising worldwide [2]. It is estimated that the overall proportion of individuals affected by HF will increase from 2.4% in 2012 to 3.0% by 2030. The prevalence of HF varies considerably, with the lowest rates found in sub-Saharan Africa. However, even in low- and middle-income countries, the prevalence is expected to rise due to population aging and the growing burden of heart failure risk factors, such as high blood pressure [3-4]. Heart failure can lead to cardiogenic shock, which is characterized by a critical state of low cardiac output that jeopardizes the perfusion and oxygenation of vital organs [5]. In-hospital mortality rates for acute heart failure range from 4% to 13%, and the mortality rate within one year after discharge is approximately 25% to 30%. Additionally, there is a significant readmission rate, with over 45% of patients requiring readmission following their initial discharge [6-7].

Current drugs are limited as they act via adrenergic receptor stimulation, which is associated with malignant arrhythmias, tachycardia, and other poor outcomes [8]. Therefore, there is an urgent need to identify new efficient inotropic modulators with a good safety profile. Istaroxime is an innovative drug that operates through a unique mechanism of action. It has a dual effect by blocking the sarcolemmal Na+/K+ pump and stimulating the activity of the sarcoplasmic reticular calcium adenosine triphosphatase isoform 2a (SERCA 2a) pump [9-10]. This dual action is achieved by displacing phospholamban from SERCA 2a, leading to improved calcium reuptake by the sarcoplasmic reticulum. Notably, this process occurs independently of the levels of cyclic adenosine monophosphate (AMP) within the cell. By lowering cytoplasmic calcium levels during the relaxation phase, istaroxime enhances cardiac relaxation, resulting in increased calcium release during contraction. Ultimately, this mechanism improves cardiac contractility [11]. Previous studies have supported the potential therapeutic role of istaroxime in the treatment of patients with acute heart failure. However, there is a lack of literature about the efficacy of istaroxime in these patients. Therefore, we have conducted this meta-analysis to assess the efficacy and safety of istaroxime in patients with heart failure.

## Review

Methodology

Following the Preferred Reporting Items for Systematic Review and Meta-Analyses (PRISMA) guidelines, a search was conducted on the EMBASE and Medline databases to identify articles related to the safety and efficacy of istaroxime in patients with heart failure. The search covered the period from inception to May 31st, 2023, without any restrictions on the year of publication. The search strategy utilized relevant terms such as "istaroxime," "heart failure," "efficacy," and other related terms, along with their corresponding Medical Subject Headings (MeSH) terms. All references were imported into EndNote X9 to remove duplicate studies. Initially, abstracts and titles of the studies were screened, followed by a detailed assessment using full-text articles. Additionally, the reference lists of all included studies were manually searched. Two authors independently performed the search and study selection. Any disagreements between the two authors were resolved through consensus or discussion with the principal investigator.

Eligibility and Selection Criteria

The inclusion criteria involved studies that assessed at least one of the outcomes considered in this meta-analysis. Only randomized controlled trials (RCTs) comparing istaroxime, at any dose, with placebo or any other drug were included. Only original studies written in English were eligible for analysis, while observational studies, systematic reviews, meta-analyses, conference abstracts, case series, correspondence, and editorials were excluded. Additionally, duplicate studies drawing conclusions from the same databases were excluded. Studies lacking a comparison group were also excluded.

Data Extraction, Outcomes, and Quality Assessment

Two authors independently extracted data from the included articles using a structured form. The extracted information included various study characteristics, such as author names, publication year, sample size, follow-up duration, and outcomes. The outcomes assessed in this meta-analysis included the change in left ventricular ejection fraction (LVEF), E to A ratio (a marker of left ventricle function), cardiac index in L/min/m2, systolic blood pressure (SBP) in mmHg, left ventricular end-systolic volume (LVESV) in ml, and left ventricular end-diastolic volume (LVDSV) in ml. For safety analysis, gastrointestinal events and cardiovascular events were assessed.

The quality of the included studies was assessed using the Cochrane Risk of Bias Assessment Tool. This tool evaluates key domains such as random sequence generation, allocation concealment, blinding of participants and personnel, blinding of outcome assessment, incomplete outcome data, selective reporting, and other sources of bias. Each domain was classified as having a low, high, or unclear risk of bias.

Statistical Analysis

The analysis was performed using RevMan Version 5.4.1 (The Cochrane Collaboration, London, United Kingdom). Pairwise meta-analysis was conducted to calculate the mean difference (MD) and the corresponding 95% confidence interval (CI) for continuous variables. For categorical variables, we calculated risk ratio (RR) with 95% CI. Statistical significance was determined for outcomes with a p-value of ≤0.05. Statistical heterogeneity was assessed using the I-squared (I2) and Cochran Q test values. An I2 value between 0% and 40% indicated low heterogeneity, while values of 30% to 60%, 50% to 90%, and 75% to 100% indicated moderate, substantial, and considerable heterogeneity, respectively. A Cochran Q test with a p-value less than 0.10 was considered significant for heterogeneity. Regardless of the heterogeneity measures, all analyses were conducted using random-effects models, as they are known to provide more robust estimates compared to fixed-effects models.

Results

**Figure 1:**
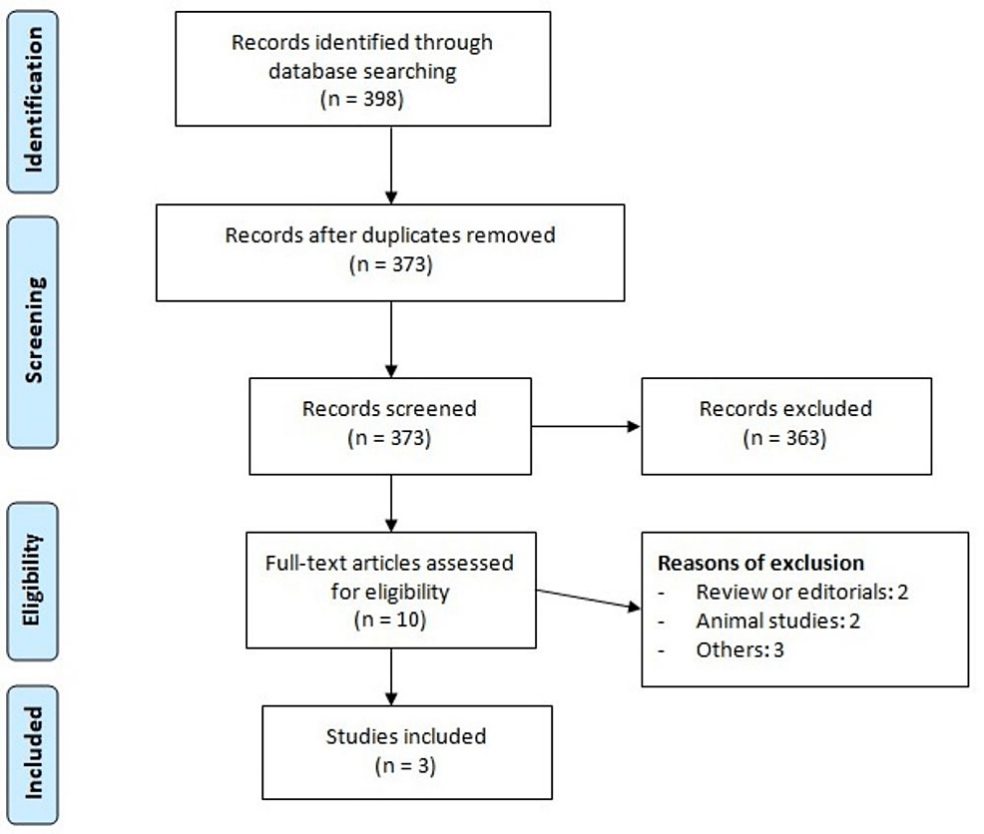
Preferred Reporting Items for Systematic Review and Meta-Analyses (PRISMA) Flowchart of Study Selection

**Figure 2:**
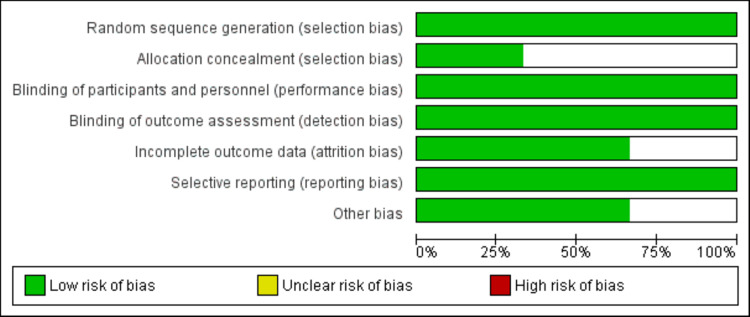
Risk of Bias Graph

Figure 1 shows the PRISMA flowchart of the study selection process. A systemic search of the online databases yielded 398 studies. After removing 25 studies, 363 studies were excluded based on study title and abstract. A total of 10 studies were selected for full-text screening, of which three RCTs were finally included in the meta-analysis. A total of 211 patients were included in our meta-analysis. Table 1 shows the characteristics of included studies. One study was analyzed twice as it analyzed two doses of istaroxime separately. The dose in included studies ranged from 1.0 to 1.5 𝛍g/kg/min. Figure 2 shows risk of bias assessment of included studies.

**Table 1:** Characteristics of Included Studies NR: Not reported

Author Name	Year	Groups	Sample Size	Dose of Istaroxime	Age (Years)	Males (%)
Carubelli et al (a) [12]	2020	Istaroxime	40	1.0 𝛍g/kg/min	52.3 vs 55.5	85 vs 90
		Placebo	20			
Gheorghiade et al (a) [13]	2008	Istaroxime	30	1.0 𝛍g/kg/min	56 vs 57	87 vs 81
		Placebo	31			
Gheorghiade et al (b) [13]	2008	Istaroxime	30	1.5 𝛍g/kg/min	54 vs 57	93 vs 81
		Placebo	31			
Metra et al [14]	2022	Istaroxime	29	1.0 𝛍g/kg/min	65.2 vs 63	76 vs 87
		Placebo	31			

LVEF (%)

**Figure 3:**
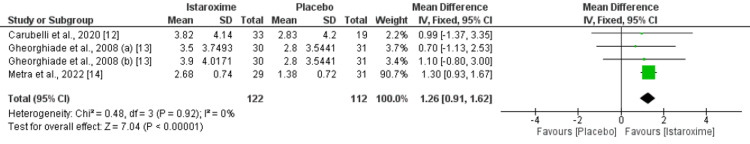
Left Ventricular Ejection Fraction (LVEF) (%) Sources: References [12-14]

**Figure 4:**
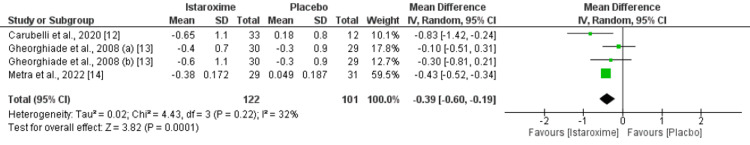
E to A Ratio Sources: References [12-14]

Increase in LVEF was significantly higher in patients receiving istaroxime than in the placebo group (MD: 1.26, 95% CI: 0.91 to 1.62, p-value: 0.001) as shown in Figure 3. No significant heterogeneity was reported among the study results (I-square: 0%). Pooled analysis of three studies showed that reduction in E to A ratio was significantly greater in patients randomized in the istaroxime group compared to the patients randomized in the placebo group (MD: -0.39, 95% CI: -0.60 to -0.19, p-value: 0.001) as shown in Figure 4. Insignificant heterogeneity was reported among the study results (I-square: 32%).

Cardiac Index (L/min/m2)

**Figure 5:**
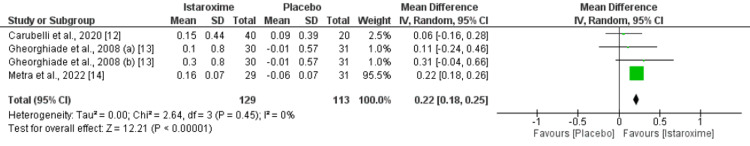
Cardiac Index (L/min/m2) Sources: References [12-14]

Pooled analysis of three studies showed that increase in cardiac index was significantly higher in patients receiving istaroxime compared to the patients in the placebo group (MD: 0.22, 95% CI: 0.18 to 0.25, p-value: 0.001) as shown in Figure 5. No significant heterogeneity was reported among the study results (I-square: 0%).

SBP (mmHg)

**Figure 6:**
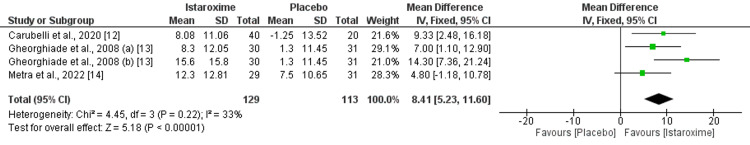
Systolic Blood Pressure (SBP) (mmHg) Sources: References [12-14]

Pooled analysis of three studies showed that increase in SBP was significantly higher in patients receiving istaroxime compared to the patients in the placebo group (MD: 8.41, 95% CI: 5.23 to 11.60, p-value: 0.001) as shown in Figure 6. No significant heterogeneity was reported among the study results (I-square: 33%).

LVESV and LVEDV (mL)

**Figure 7:**
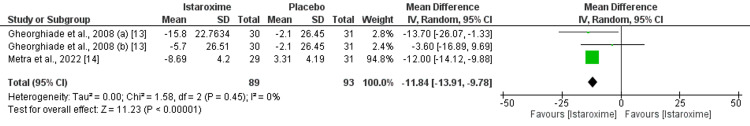
Left Ventricular End-Systolic Volume (LVESV) (mL) Sources: References [13-14]

**Figure 8:**
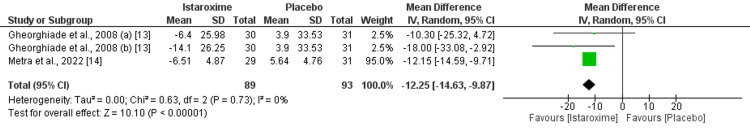
Left Ventricular End-Diastolic Volume (LVDSV) (mL) Sources: References [13-14]

Pooled analysis of two studies showed that that reduction in LVESV is significantly higher in patients receiving istaroxime group compared to the patients in the placebo group (MD: -11.84, 95% CI: -13.91 to -9.78, p-value: 0.001, I-square: 0%) as shown in Figure 7. Similarly, reduction in LVEDV is significantly higher in patients receiving istaroxime group compared to the patients in the placebo group (MD: -12.25, 95% CI: -14.63 to -9.87, p-value: 0.001, I-square: 0%) as shown in Figure 8.

Safety Analysis

**Figure 9:**
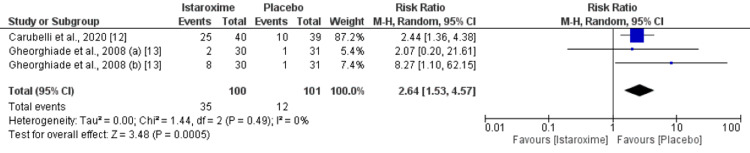
Gastrointestinal Events Sources: References [12-13]

**Figure 10:**
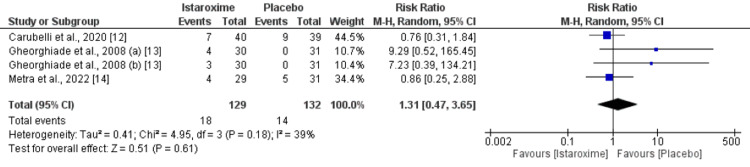
Cardiovascular Events Sources: References [12-14]

As shown in Figure 9, risk of gastrointestinal events was significantly higher in patients receiving istaroxime compared to the placebo group (RR: 2.64, 95% CI: 1.53 to 4.57, p-value: 0.0005). However, risk of cardiovascular events was not different between the two groups (RR: 1.31, 95% CI: 0.47 to 3.65, p-value: 0.61) as shown in Figure 10.

Discussion

This meta-analysis assessed the efficacy and safety of istaroxime in patients with heart failure. The pooled analysis of three RCTs showed that infusion of istaroxime is associated with a better efficacy profile, including a greater reduction in the E to A ratio, LVESV, and LVEDV. Additionally, we also found that the increase in cardiac index, ejection fraction, and SBP was significantly higher in the istaroxime group compared to the placebo group within 24 hours of infusion. The results indicate that istaroxime enhances heart function in this group of patients, which aligns with earlier clinical and experimental evidence [15]. The simultaneous rise in cardiac index and blood pressure sets it apart from previous intravenous medications given to patients, offering the possibility of quicker stabilization and earlier implementation of other life-saving treatments. This aligns with the pharmacokinetic characteristics of istaroxime, which exhibit a swift onset of effect and a comparably rapid elimination after the infusion ends [16]. These attributes make istaroxime a viable option for administering to high-risk individuals and potentially to those in critical condition.

Istaroxime functions as an inhibitor of the sodium-potassium adenosine triphosphatase pump while also enhancing the activity of SERCA 2a [17]. The idea that improving SERCA 2a activity could be a promising therapeutic approach for acute heart failure is supported by positive outcomes observed in animal models involving either SERCA 2a overexpression or reduced phospholamban activity [18]. Istaroxime exhibits a more pronounced effect on sodium-potassium pump inhibition compared to digoxin. Laboratory experiments conducted with guinea pigs and dogs, both in vitro and in vivo, have demonstrated dose-dependent increases in inotropic activity measured by the derivative of the maximum rate of left ventricular pressure increase (dP/dtmax) in response to istaroxime therapy [17]. In a chronic model of canine heart failure, administration of istaroxime led to dose-dependent reductions in left ventricular end-diastolic and end-systolic volumes and a significant increase in left ventricular ejection fraction, without elevating myocardial oxygen consumption [19]. These findings are consistent with the results reported in our study, demonstrating the efficacy of istaroxime in patients with heart failure.

While other inotropes may enhance cardiac performance to a similar or greater degree compared to istaroxime, the distinguishing factor of this medication lies in its impact on blood pressure and heart rate. It is noteworthy that istaroxime led to a decrease in E/A (a marker of diastolic function) while simultaneously increasing SBP, which is remarkable since elevated afterload has typically been associated with decreased E' [20]. Considering the point that istaroxime has been reported to increase SBP, which can be beneficial in certain situations where low blood pressure is a concern, dobutamine, on the other hand, may cause a decrease in systemic vascular resistance, potentially leading to a drop in blood pressure [21]. Therefore, for patients with heart failure and low systolic blood pressure, istaroxime is beneficial. Further research studies with larger sample sizes and/or longer durations of istaroxime infusion are needed to fully understand this finding.

Treatment with istaroxime was associated with more adverse events, including gastrointestinal issues, compared to the placebo. However, the risk of cardiovascular events was not significantly different between the two groups. Several strategies have been explored to mitigate gastrointestinal side effects and injection site pain associated with istaroxime. One approach involves the development of a liposomal formulation of istaroxime, where the drug is encapsulated in a specially designed drug delivery system that quickly disintegrates in plasma. This approach aims to minimize any potential impact on the pharmacokinetic profile of istaroxime. To achieve this, an excipient called polyethylene glycol 660-hydroxystearate (PEG-HS) was chosen to modulate the fluidity of the liposome's bilayer and control its release properties. The result was an almost complete release of istaroxime within physiological conditions in less than 10 minutes, thereby addressing concerns related to gastrointestinal adverse effects [22].

To our knowledge, this is the first meta-analysis to date providing a comprehensive analysis of the efficacy and safety of istaroxime in patients with heart failure. However, the current meta-analysis has certain limitations. Firstly, only three RCTs were included in this study with a pooled sample size of 211. Secondly, we were not able to perform subgroup analysis due to lack of individual-level data. Lastly, the follow-ups of the studies were 24 hours, so long-term impacts were not reported. Hence, more studies are required to further analyze the short-term and long-term beneficial effects of istaroxime in comparison with other inotropic drugs.

## Conclusions

In conclusion, this meta-analysis demonstrates that istaroxime is associated with improved efficacy in patients with heart failure, as indicated by increased cardiac index, ejection fraction, and systolic blood pressure, as well as reduced E to A ratio, left ventricular end-systolic volume, and left ventricular end-diastolic volume. These findings support the enhancement of heart function with istaroxime administration, aligning with previous clinical and experimental evidence. Istaroxime's unique ability to simultaneously increase cardiac index and blood pressure sets it apart from other intravenous medications used in patients with heart failure, offering the potential for quicker stabilization and earlier implementation of life-saving treatments. In terms of safety, istaroxime treatment was associated with a higher risk of gastrointestinal events compared to the placebo, while the risk of cardiovascular events did not significantly differ between the two groups. Further investigations are necessary to optimize the safety profile of istaroxime.
